# The effect of introducing a free breakfast club on eating habits among students at vocational schools

**DOI:** 10.1186/s12889-019-6701-9

**Published:** 2019-04-03

**Authors:** Camilla Berg Christensen, Bent Egberg Mikkelsen, Ulla Toft

**Affiliations:** 1 Department for Development and Planning, Aalborg University Copenhagen, A. C. Meyers Vænge 15, DK-2450 Copenhagen, SV Denmark; 2 Department of Education, Learning and Philosophy, Aalborg University, A. C. Meyers Vænge 15, DK-2450 Copenhagen, SV Denmark; 3 Centre for Clinical Research and Prevention Bispebjerg and Frederiksberg Hospital, Hovedvejen, Entrance 5, Nordre Fasanvej 57, DK-2000 Frederiksberg, Denmark

**Keywords:** Randomised controlled trial, Low socioeconomic status, Breakfast club, School meals, Adolescents, Eating habits, Health promotion

## Abstract

**Background:**

Unhealthy eating habits are a major problem among adolescents. The objective of the study was to assess the effect of a free breakfast club intervention on dietary habits among students at vocational schools.

**Methods:**

The study included students (*n* = 318) from four vocational schools in Denmark. Food frequency questionnaires were used to measure eating habits at baseline, first, and second follow-up, after 7 and 14 weeks respectively, in a clustered randomized controlled intervention of four months. The effect of the intervention was evaluated through self-reported frequencies of breakfast intake, intake of whole grain products for breakfast and intake of unhealthy snacking in the morning. The outcome measures were daily breakfast intake (yes/no), daily intake of whole grain for breakfast (yes/no), and unhealthy snacking on school day mornings (yes/no).

**Results:**

The proportion of students who had breakfast every school day increased significantly in the intervention schools from baseline to the first follow-up compared to the control group (OR: 3.77; *P* = 0.0149). The effect was attenuated at the second follow-up. The intake of whole grain products for breakfast increased significantly more among students in intervention schools compared to students in control schools both at first (OR: 4.13; *P* = 0.0079) and second follow-up (OR: 3.27; *P* = 0.0317). No significant change in unhealthy snacking was found.

**Conclusion:**

Provision of free breakfast at vocational schools can improve the dietary quality of breakfast and decrease breakfast skipping. However, the sustainability of the intervention is a critical issue that needs to be further studied and addressed.

**Trial registration:**

ISRCTN11265280. Registered 20 November 2018 (retrospectively registered).

**Electronic supplementary material:**

The online version of this article (10.1186/s12889-019-6701-9) contains supplementary material, which is available to authorized users.

## Background

Breakfast is commonly recognized as an important meal to maintain good eating patterns and an essential source of macro- and micronutrients. Comparisons between breakfast eaters and breakfast skippers have shown that breakfast eaters are more likely to meet micronutrient recommendations than breakfast skippers [1–3]. In general, breakfast cereals provide a higher level of fibre, iron, folic acid, and zinc relative to a non-cereal breakfast [4, 5]. Moreover, whole grain (WG) cereals contain key components that are associated with protective effects against certain types of cancer, cardiovascular disease, diabetes, and obesity [6–8]. Considering these beneficial effects, a worrying portion of young people skips breakfast [3, 9, 10]. An American study reported that 31.5% of adolescents were breakfast skippers [3]. Furthermore, a cross-sectional Dutch study of youngsters in different subgroups, all attending school, showed that between 62.9 and 95.5% consumed breakfast every day [11].

Further, breakfast skipping is not evenly distributed across the population. Adolescents and population groups with lower socioeconomic status (SES) are less likely to regularly eat breakfast compared to those with higher SES [12, 13] and fewer meet the recommendations for WG intake [14, 15]. The National Food Institute in Denmark has reported that 27% of the population eats the recommended WG amount of at least 75 g of whole grain per 10 megajoules per day [16]. The same report recommends that increased intake can be reached through the breakfast meal [16]. Additionally, eating habits acquired before and in adolescence seem to track into adulthood [17, 18]. It is therefore highly relevant to promote good eating habits among adolescents and young people, especially those belonging to the vulnerable population groups. Vocational schools provide a good opportunity to influence the eating habits of young people with low SES, such as those training for occupations as motor mechanics, carpenters, and electricians. Since the Ottawa Charter for Health Promotion, it has been realized that the environments where people live influence their behaviour considerably [19]. As a result, there is a strong rationale for intervening in the settings of everyday life by providing opportunity for healthy food choices. Following this, one way to increase opportunity and thereby affect the eating habits of young people is to increase the availability of healthy whole grain breakfast, for example in breakfast clubs. The concept of breakfast clubs, where schools serve breakfast to their students, was originally introduced as a strategy to provide a healthy meal to disadvantaged groups [20]. In order to increase the likelihood of making health promotion interventions persist beyond the intervention period, it is suggested that the intervention should preferably adapt the local context in which it is implemented [21]. This study implements a breakfast club under controlled program conditions but in a “real world” setting and where it is possible to adapt to the context.

Breakfast clubs have been most commonly used in primary school settings and more seldom in the setting of secondary schools, e.g., vocational schools [9, 22]. In addition, studies are lacking on the effects of breakfast club intervention (BCI) based on WG products. The aim of this study was to investigate the effects of a BCI in a vocational school setting for adolescents by assessing selected eating habits among subjects exposed to a breakfast club intervention compared to schools without a BCI. In particular, the aim was to investigate whether the implementation of a BCI had an effect on the frequency and quality of breakfast eating of students and if there was an effect on the degree of unhealthy snacking in the morning.

## Methods

### Study design

School recruitment was established with a call for candidate schools. Schools fulfilling a set of eligibility criteria were interviewed and the final selection was made. Eligibility criteria for the schools to be part of the intervention were:

- Willingness to serve whole grain breakfast during first lesson to a proportion of their students.
- Willingness to follow either control or intervention arms of the study, depending on the randomization.
- Willingness to pay expenses, e.g., extra cleaning, service, and milk connected to implementation of the BCI.
- Provision of vocational training for students wishing to become electricians or motor mechanics.
- Not currently providing a similar health intervention involving breakfast.

Four schools were included in the intervention and were randomly assigned to either an intervention or a control arm. Two of the schools (A and C) were allocated from rural areas and two (B and D) were allocated from urban areas. The latter schools were located at two different sites but belonged to the same institution. To ensure both a rural and an urban school in the intervention/control group a rural and an urban located school were randomly combined. Afterwards these blocks were randomly selected for either control or intervention by an independent co-worker that allocated schools (cluster level) by the means of concealed envelopes. To ensure willingness to participate in randomization with the chance of not serving breakfast to students, control schools received breakfast after the intervention had finished, from August 2012.

The study was performed in accordance with the guidelines of the Declaration of Helsinki and complied with the regulations on nonclinical trials by the National Committee on Health Research Ethics in Denmark [23, 24]. Participants were all older than 16 years of age. Written consent was received through answering the questionnaire. As such, I informed participating students, in writing through a participant information sheet and orally, about the purpose of the study, the use of data and that answering questionnaire was voluntary. The survey did not require approval from Data Protection Agency, which was confirmed by the Aalborg University Data Protection Unit. The current study is registered at the Aalborg University Contract Unit under the id: 2018–899/10–0068. The study adheres to CONSORT guidelines.

**Fig. 1:**
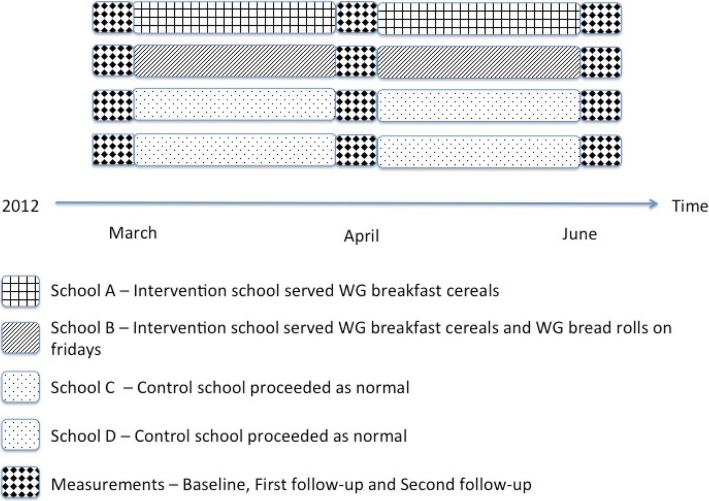
Study design after randomization with the four participating schools and the three measurements

To implement the BCI, intervention schools served a free whole grain breakfast every school day as part of the first lesson; this occurred either in the classroom or in a small cafeteria. The breakfast consisted of a choice among four WG cereal products. The cereals contained between 80 and 100% of WG. One school served bread rolls once a week (Friday); the bread rolls contained more than 50% of WG. All products met the guidelines for WG content in food products as stated by the National Food Institute [16]. Control schools carried on as normal without the availability of free breakfast. The duration of the intervention was approximately four months, from early March to late June in 2012. The study design encompassed three measurements: baseline, performed just before the initiation of the intervention, in week 0 (February 27–March 2); first follow-up in week 7 and 8 after baseline (April 16–27) and second follow-up performed from week 14 to 16 after baseline (June 4–22). Figure 1 shows the design of the study with timeline, measurements, and school allocation to either control or intervention. All measurements occurred before lunch and during class. The researcher was present during measurements and introduced the questionnaire to the students, collectively in class, before the questionnaires were handed out. Additionally, the researcher remained available to answer questions or provide assistance and also collected the questionnaires after completion.

### Subjects

Student participants attended one of four vocational schools that are within the Danish vocational education and training programmes. The programmes, between one and one-half years and five years in length, consist of a basic and a main course; in addition to completing classroom education, students must attain competence within an internship in a business. A total of 111 vocational specializations are available, of which areas such as building/construction and electricity/management/IT are typical [25]. To produce a homogenous study sample, recruitment was targeted to students from educational programs that were common in the included schools. Overall, the sampling can be characterised as intentional but with elements of convenience sampling [26] in the sense that it depended on students’ willingness to enrol voluntarily. The study included 318 students. The study population was not entirely uniform at the three measurements. The number participants at each measurement are illustrated in Table 1. Although subjects’ names were indicated on questionnaires associated with the three measurements, in order to track students over time, names were removed during data processing to ensure confidentiality.

**Table 1:** Number of participants at baseline, first and second follow-up

	Number of participants		
Baseline	252		
First follow-up		168	
Second follow-up			104

A total of 318 participants were included in the study with variant degree of participation. 165 participants solely participated in one measurement, 100 participated in two measurements and 53 participated in all three measurements

Basic characteristics of the participating students are given in Table 2.

**Table 2:** Demographic characteristics of students at baseline divided into control or intervention group

	Control	Intervention	*P* value*
	N (%)	N (%)	
Age			0.3446
[16–19]	62 (24.7)	76 (30.3)	
[20–24]	38 (15.1)	36 (14.3)	
[25–29]	12 (4.8)	12 (4.8)	
[30 –)	4 (1.6)	11 (4.4)	
Gender			0.9587
Male	111 (44.2)	129 (51.4)	
Female	5 (2.0)	6 (2.4)	
Education, type of program enrolled in			< 0.0001*
Car, airplane, and transportation	0	62 (24.6)	
Production and development	4 (1.6)	0	
Electricity, management, and IT	91 (36.1)	74 (29.4)	
Transport and logistics	21 (8.3)	0	
Housing conditions			0.3698
Live alone	23 (9.2)	19 (7.6)	
With one or both biological parents	68 (27.2)	72 (28.8)	
With spouse/girlfriend/boyfriend or children	14 (5.6)	28 (11.2)	
With a friend	4 (1.6)	5 (2.0)	
Dormitory, boarding school, or house share	2 (0.8)	5 (2.0)	
Other adults	4 (1.6)	6 (2.4)	

There are missing values in these characteristic frequencies due to missing answers in the particular questions

*Tested by Chi-square test

### Questionnaire

Dietary intake was measured by questionnaire and included questions on frequency of breakfast intake, type of breakfast, and frequency of snacking unhealthy food in the mornings. The questionnaire comprised 29 questions that encompassed demographic information, behavioural involvement, and attitudinal questions. Questions were divided into seven sections: demographic information, transportation to school, breakfast habits, morning eating habits, breakfast served at school, well-being in school, and food habits in general (Additional file 1). Here we focus on the questions that elucidate the study’s aim.

The section on breakfast habits included six questions, of which two were used in this paper. Besides breakfast habits unhealthy snacking in the morning was included in the paper. The breakfast habit section began by defining breakfast as food or drinks consumed before 9 AM. Breakfast consumption was self-reported via a frequency scale and was formulated as a recall question of the previous week’s total breakfast consumption during weekdays. The scale was: all 5 days, 3–4 days, 1–2 days, and 0 days. In the same section, a breakfast food frequency question assessed the whole grain content of students’ breakfast eating habits, focusing on intake in the prior school week. Choices included 13 common types of breakfast possibilities, with a frequency scale of four possibilities ranging from all five school days to none. Consequently, these two questions were considered applicable to investigate the research aim, which was to elucidate whether the BCI had an impact on dietary habits compared to no intervention. Dietary habits were assessed through collection of data on regularity of breakfast intake, quality of breakfast (interpreted as containing WG), and whether unhealthy snacking was affected.

Furthermore, students were requested to recall their level of snacking on school day mornings (i.e., 9–11 AM) in the previous week, marking 11 types of snacks on a frequency scale (all 5 days, 3–4 days, 1–2 days, 0 days). Overall, food and beverage items included in the food frequency questions were informed by previous reports about what constitutes contemporary eating habits in the Danish population among children and young people (4–24 years old), and short-educated adults, where the highest completed education is primary school or vocational education [27, 28].

The questionnaire, which was developed for this study was inspired by two similar questionnaires [29, 30]. And was pretested among a group of six students attending a vocational school and subsequently modified to achieve more clarity and avoid possible misinterpretations.

### Dietary outcomes

The selected dietary habit outcomes were all dichotomized. The question concerning frequency of breakfast intake was recoded into daily breakfast eaters if subjects consumed breakfast all school days (yes/no).

Similarly, to assess the intake of WG breakfast products, a food group of WG products was created among several food items. Thus, students were divided into two categories, yes or no, for eating either WG cereals or WG bread at five school days. Unhealthy snacking habits in the morning were defined by the intake of candy, chocolate, chips, fast food, cake, crackers, or Danish pastries before lunch. A variable for unhealthy snacking was defined as the intake of these five items, for all five school days a week, as a yes/no construct.

### Missing data

The questionnaire response rate was 98%. Seven questionnaires were not filled out and excluded from analysis. If participants reported an intake of foods in a category but answers were missing for other food items in the same category, the answers for these food items were interpreted as “never”.

### Statistical analyses

All analyses were done with an intention-to-treat approach. Intention-to-treat analysis is relevant because it includes all participants in both the intervention and control groups regardless of whether they accepted or adhered to the intervention (i.e., if they actually ate the breakfast served) [31]. SAS V9.3 (Cary, North Carolina, USA) software was used to perform the statistical analyses. They included descriptive statistics of respondents at baseline. Further, the fitted generalized linear mixed model (SAS PROC GLIMMIX procedure) was used to analyse whether there was a significant difference between control and intervention schools over time in regard to the main outcomes. For all GLIMMIX procedures, the fixed effects were condition and time (time of measurement) and their interactions. Random effects were school, id, school*time, and id*time.

## Results

The descriptive statistics at baseline showed that there was significant difference (*p* < 0.05) between control and intervention group in education type of programme the students were enrolled in. There were no statistically differences in other descriptive variables of participants (Table 2). At the first follow-up, there was a significant increase in the proportion of students who had breakfast (daily breakfast eaters) in the intervention group (OR: 3.77; *P* = 0.0149) compared to the control group. At the second follow-up, the effect attenuated and was no longer significant. The overall development during the whole intervention period was nonsignificant (*P* = 0.1657).

There were significant increases in the proportion of students who had WG products for breakfast in the intervention group compared to the control group. These positive significant differences were found both at the first follow-up (OR: 4.13; *P* = 0.0079) and at the second follow-up (OR: 3.27; *P* = 0.0317). However, the overall trend during the whole intervention period was nonsignificant.

No significant differences were found across the study period in snacking on unhealthy foods in the morning (data not shown).

The used fitted generalized linear mixed model is able to analyse longitudinally studies with repeated measures over time that contain both independent and not independent observations; this capability includes, as for this study, analysis of the same individual with repeated measures that are not uniform throughout measurements [32]. Additionally, the model takes clustered sampling into account (e.g., students who attend the same school have common characteristics).

## Discussion

Results from this study showed significant beneficial effects of a BCI on quality of breakfast intake and breakfast skipping among students at vocational schools. No associated effect was found regarding unhealthy snacking behaviour in the morning.

However, the study showed that the persistence of the change in daily breakfast intake frequency was weak and not significant over the entire intervention period. The persistence might be improved by a whole-school approach, or it might require political prioritization and external funding, because most vocational schools currently have insufficient funds to implement such a program. The lack of persistent effects was also seen in a study by Ask et al. that was performed in a lower secondary school. The 54 students in the study received one hour of information about the importance of a healthy diet, and an intervention arm received free breakfast and food supplements for four months. Meal patterns, measured by a food frequency questionnaire, showed that almost all students had breakfast at school while the intervention lasted. However, one week post-intervention, all students in the intervention group had returned to their normal breakfast pattern [33]. Eating habits are complex, and it is proposed that they extend beyond repeated behaviour. Establishing and changing existing habits may need elements such as situational factors and self-regulation skills to be effective [34]. The challenge of changing sustained eating habits may lie behind the non-persistent breakfast eating behaviour. The attenuation in the proportion of daily breakfast eaters could also be explained by the bias sometimes referred to as the Hawthorne effect, where an intervention affects subjects’ behaviour due to their participation rather than the intervention per se [35]. In this case, an initial interest was seen among the students when the breakfast club was first implemented, yet this effect faded and thus the proportion of daily breakfast eaters was reduced.

Students in intervention schools, in the present study, significantly increased their intake of WG products. These findings are concordant with results from a study of the impact of a national free healthy breakfast program [36]. Students (9–11 years) in 111 primary schools in Wales were enrolled in a clustered randomized controlled trial with repeated cross-sectional design and a 12-month follow-up. Intervention schools served a healthy breakfast before school. The results showed that children from schools that served breakfast ate significantly more healthy food items (cereal, bread, milk, and fruit) for breakfast compared to control schools. Although the study by Murphy et al. is different from the present study in numerous ways, the results of healthier breakfast intake are parallel. Accordingly, the intake of WG products during breakfast at both the first and second follow-up was higher in the intervention group than in the control. It may be that members of the intervention group have substituted some of their usual (not WG) breakfast with whole grain breakfast and this is reflected in the attenuation of breakfast intake frequency and the sustained difference in WG intake. However, the length of the intervention is probably not long enough to establish a permanent change in eating habits.

Compliance with dietary guidelines is recommended to gain a balanced diet and lower the risk of developing non-communicable diseases. The Danish dietary guidelines contain 10 recommendations, e.g., “Choose whole grains” and “Eat more fish”, and each recommendation is accompanied with more detailed recommendations and suggestions for how to achieve the goal [37]. The Danish recommendations for a healthy breakfast meal are in accordance with the overall guidelines and suggested to contain fruit or vegetables, WG bread, or WG cereal products supplemented by skimmed milk or low-fat dairy products [38]. In the present intervention, schools offered breakfast meals that were in line with the Danish recommendations regarding WG, and through this intervention they potentially increased WG intake among students. However, because the main focus was on a single component in the dietary guidelines, it could be argued that the intervention under-prioritized other healthy foods.

The longitudinal design strengthened the present study by allowing for exploration of the development of food habits over time in the BCI. The addition of a measurement in the middle of intervention (between baseline and post-intervention) provided an opportunity to explore whether there was an initial effect that attenuated in the second follow-up.

Questionnaires were handed out and completed during class, where the majority of students were present and given time to respond. In this way a high response rate was achieved. Another strength was that the researcher was present to assist students who had questions about completing the questionnaire, and the researcher also could detect incomplete answers when the questionnaires were collected. This kept missing values to a minimum and ensured a more complete data set.

Although food frequency questionnaires have been accepted as a method for estimation of intake, its shortcomings are well discussed in the literature [39, 40]. Due to time and financial constraints, the questionnaire was not validated, an omission that carries the risk of the questionnaire not measuring what was intended. To compensate, the questionnaire was pretested. The chosen procedure to replace missing values may have introduced bias because the true value is unknown and the substitution can alter statistical results [41].

As stated in a report from the Danish Ministry of Education [42], Danish vocational and educational programmes are in general gender segregated. In the same report figures, from 2015, show that the area of technology, building, construction, and transportation was attended by 30,038 males but only 3448 females (88.53 and 11.47%, respectively), while health care and pedagogy was attended by 2481 males and 15,916 females (15.59 and 84.41%, respectively) [42]. The gender segregation is also illustrated in the present study, in which the majority of participants were male (95.6%) (Table 2). This is due to the traditional male predomination in the chosen programmes. Because the sample does not resemble the general population or other types of educational programmes (e.g., health care and pedagogy), this is a study limitation that limits its external validity.

The statistical analysis would have been stronger if repeated measurements had been performed in a larger sample. Although agreement about measurement times was reached with school managers prior to study initiation, it was difficult to obtain repeated measurements. This affected the type of statistical analysis chosen (i.e., the GLIMMIX procedure). Because participant groups were predetermined, a statistical power calculation was not performed due to the practical limitations of expanding the sample. Finally, differences were significant between each measurement and the baseline, but the overall trend was not significant. Hence, results need to be interpreted carefully.

There was a statistical difference in baseline descriptive statistics as students in control and intervention groups were enrolled in different types of educational programmes. This difference may have had an impact on the results as students attending different programmes may have different breakfast habits.

School managers, policy makers, and NGOs can benefit from the results of the study, which reveals strategic opportunities to improve health through the provision of healthy breakfasts in school settings. School management has the authority to improve food policies in the schools. Policy change might have the ability to improve the eating environment and produce healthier eating habits [43]. One challenge, however, is that most schools are located in areas with purchase opportunities in the surrounding environment. To gain knowledge about possibilities for creating change, it is essential to take a holistic approach, where surrounding environments are included. The government has the opportunity to prioritize healthy eating surroundings in school settings and to provide essential funds to schools to implement changes. NGOs should exploit openings to achieve a healthier lifestyle for all young people regardless of socioeconomic status. As shown by O’Loughlin et al. [44], it is challenging to achieve persistence in interventions. The Canadian study investigated a total of 189 health promotion interventions and found that six in 10 did not achieve permanent sustainability. The authors further suggested that to obtain sustainability in interventions, and perhaps sustained change in behaviour, certain factors seem to be important. These include staff who work without payment and the presence of program champions [44]. This might be obtainable through collaboration between a NGO and local champions. A longitudinal study in which the breakfast club’s effects on eating habits are followed for a longer time would benefit research. It is critical to elucidate the breakfast club’s ability to even out the inequity in health-related behaviours that are related to socioeconomic status.

## Conclusion

In conclusion, the present study showed that introducing a BCI improved the quality of breakfast intake and reduced breakfast skipping among students at vocational schools. No effect was found on snacking behaviour in the morning. Breakfast clubs provide a strategy that authorities may use to improve the eating habits of population groups that are prone to unhealthy eating habits, especially young people with low SES. This study contributes to the research about breakfast availability in schools in a fairly unstudied population group. The results suggest that breakfast clubs may be a public health strategy to improve eating habits among young people from low SES groups. However, more research is needed to find effective ways to improve the sustainability of such interventions.

## Additional file

Additional file 1: Questionnaire to students The questionnaire concerns eating habits and students weekdays in school. (DOCX 88 kb)

## Abbreviations

BCI: Breakfast club intervention
SES: Socioeconomic status
WG: Whole grain

## References

[1] Fayet-Moore F, Mcconnell A, Tuck K, Petocz P (2017). Breakfast and breakfast cereal choice and its impact on nutrient and sugar intakes and anthropometric measures among a nationally representative sample of Australian children and adolescents. Nutrients.

[2] Rampersaud GC, Pereira MA, Girard BL, Adams J, Metzl JD (2005). Breakfast habits, nutritional status, body weight, and academic performance in children and adolescents. J Am Diet Assoc.

[3] Deshmukh-Taskar P, Nicklas TA, O'Neil CE, Keast DR, Radcliffe JD, Cho S (2010). The relationship of breakfast skipping and type of breakfast consumption with nutrient intake and weight status in children and adolescents: the National Health and nutrition examination survey 1999-2006. J Am Diet Assoc.

[4] Albertson AM, Thompson D, Franko DL, Kleinman RE, Barton BA, Crockett SJ (2008). Consumption of breakfast cereal is associated with positive health outcomes: evidence from the National Heart, Lung, and Blood Institute growth and health study. Nutr Res.

[5] Tolfrey K, Zakrzewski JK (2012). Breakfast, glycaemic index and health in young people. JSHS.

[6] Egeberg R, Olsen A, Loft S, Christensen J, Johnsen N, Overvad K, Tjønneland A (2010). Intake of wholegrain products and risk of colorectal cancers in the diet, Cancer and health cohort study. Br J Cancer.

[7] Parker ED, Liu S, Van Horn L, Tinker LF, Shikany JM, Eaton CB, Margolis KL (2013). The association of whole grain consumption with incident type 2 diabetes: the Women's Health Initiative observational study. Ann Epidemiol.

[8] Aune D, Keum N, Giovannucci E, Fadnes LT, Boffetta P, Greenwood DC, Tonstad S, Vatten LJ, Riboli E, Norat T (2016). Whole grain consumption and risk of cardiovascular disease, cancer, and all cause and cause specific mortality: systematic review and dose- response meta- analysis of prospective studies. BMJ.

[9] Alexy U, Wicher M, Kersting M (2010). Breakfast trends in children and adolescents: frequency and quality. Public Health Nutr.

[10] Dwyer JT, Evans M, Stone EJ, Feldman HA, Lytle L, Hoelscher D, Johnson C, Zive M, Yang M (2001). Adolescents’ eating patterns influence their nutrient intakes. J Am Diet Assoc.

[11] Raaijmakers LGM, Bessems KMHH, Kremers SPJ, van Assema P (2010). Breakfast consumption among children and adolescents in the Netherlands. Eur J Pub Health.

[12] Vereecken C, Dupuy M, Rasmussen M, Kelly C, Nansel TR, Al Sabbah H, Baldassari D, Jordan MD, Maes L, Niclasen BV (2009). Breakfast consumption and its socio-demographic and lifestyle correlates in schoolchildren in 41 countries participating in the HBSC study. Int J Public Health.

[13] Höglund D, Samuelson G, Mark A (1998). Food habits in Swedish adolescents in relation to socioeconomic conditions. Eur J Clin Nutr.

[14] Alexy U, Zorn C, Kersting M (2010). Whole grain in children's diet: intake, food sources and trends. Eur J Clin Nutr.

[15] Kyrø C, Skeie G, Dragsted LO, Christensen J, Overvad K, Hallmans G, Johansson I, Lund E, Slimani N, Johnsen NF, HalkjæR J, Tjønneland A, Olsen A (2012). Intake of whole grain in Scandinavia: intake, sources and compliance with new national recommendations. Scand J Public Health.

[16] Mejborn H, Biltoft-Jensen A, Trolle E, Tetens IF. Fuldkorn - Definition og vidensgrundlag for anbefaling af fuldkornsindtag i Danmark 2008. 1–90.

[17] Kaikkonen JE, Mikkilä V, Magnussen CG, Juonala M, Viikari JS, Raitakari OT (2013). Does childhood nutrition influence adult cardiovascular disease risk? Insights from the young Finns study. Ann Med.

[18] Lake AA, Adamson AJ, Craigie AM, Rugg-Gunn AJ, Mathers JC (2009). Tracking of dietary intake and factors associated with dietary change from early adolescence to adulthood: the ASH30 study. Obesity Facts.

[19] Kickbusch I (2003). The contribution of the World Health Organization to a new public health and health promotion. Am J Public Health.

[20] Basch CE (2011). Breakfast and the achievement gap among urban minority youth. J Sch Health.

[21] Whelan J, Love P, Pettman T, Doyle J, Booth S, Smith E, Waters E (2014). Cochrane update: predicting sustainability of intervention effects in public health evidence: identifying key elements to provide guidance. J Public Health.

[22] Hoyland A, Dye L, Lawton CL (2009). A systematic review of the effect of breakfast on the cognitive performance of children and adolescents. Nutr Res Rev.

[23] Sundheds- og Ældreministeriet. Lov om videnskabsetisk behandling af sundhedsvidenskabelige forskningsprojekter. 2011, Lov nr 593 af 14/06/2011. Accessed 10 Oct 2014.

[24] World Medical Association (2013). World medical association declaration of Helsinki: ethical principles for medical research involving human subjects. JAMA.

[25] Vocational education and training (vet). http://eng.uvm.dk/upper-secondary-education/vocational-education-and-training-in-denmark-. Accessed 17 Oct 2017.

[26] Hultsch DF, Hunter MA, Maitland SB, Dixon RA (2002). Sampling and generalisability in developmental research: comparison of random and convenience samples of older adults. Int J Behav Dev.

[27] Christensen LM, Kørup K, Trolle E, Fagt S. Mænds måltidsvaner, viden om og holdninger til at spise sundt i forhold til uddannelse 2011–2013. 2015. 3–42.

[28] Fagt S, Christensen T, Groth MV, Biltoft Jensen A, Matthiessen J, Trolle E (2000). Børn og unges måltidsvaner.

[29] Øe K, Ilsøe A, Arentsen I (2011). Kostundersøgelse på erhvervsuddannelserne.

[30] De Bourdeaudhuij I, Klepp K, Due P, Rodrigo CP, De Almeida M, Wind M, Krolner R, Sandvik C, Brug J (2005). Reliability and validity of a questionnaire to measure personal, social and environmental correlates of fruit and vegetable intake in 10-11-year-old children in five European countries. Public Health Nutr.

[31] Kirkwood BR, Sterne JA. Linking analysis to study design: summary of methods. In Medical statistics. second edition. Edited by Goodgame F, Pinder V, Moore K. Oxford: Blackwell Publishing Ltd; 2003:395–412

[32] Cheng J, Edwards LJ, Maldonado-Molina MM, Komro KA, Muller KE (2010). Real longitudinal data analysis for real people: building a good enough mixed model. Stat Med.

[33] Ask AS, Hernes S, Aarek I, Johannessen G, Haugen M (2006). Changes in dietary pattern in 15 year old adolescents following a 4 month dietary intervention with school breakfast – a pilot study. Nutr J.

[34] Jv R, Sijtsema SJ (2011). Dagevos H, De Bruijn G. The importance of habits in eating behaviour. An overview and recommendations for future research. Appetite.

[35] Wickström G, Bendix T (2000). The" Hawthorne effect"—what did the original Hawthorne studies actually show?. Scand J Work Environ Health.

[36] Murphy S, Moore GF, Tapper K, Lynch R, Clarke R, Raisanen L, Desousa C, Moore L (2011). Free healthy breakfasts in primary schools: a cluster randomised controlled trial of a policy intervention in Wales. UK Public Health Nutr.

[37] Montagnese C, Santarpia L, Buonifacio M, Nardelli A, Caldara AR, Silvestri E, Contaldo F, Pasanisi F (2015). European food-based dietary guidelines: a comparison and update. Nutrition.

[38] Alt om Kost. http://altomkost.dk/fileadmin/user_upload/altomkost.dk/Maaltidsmaerket/Guide_skole.pdf. Accessed 14 June 2017.

[39] Cade J, Burley V, Warm D, Thompson R, Margetts B (2004). Food-frequency questionnaires: a review of their design, validation and utilisation. Nutr Res Rev.

[40] Araujo MC, Yokoo EM, Pereira RA (2010). Validation and calibration of a semiquantitative food frequency questionnaire designed for adolescents. J Am Diet Assoc.

[41] Parr CL, Hjartåker A, Scheel I, Lund E, Laake P, Veierød M (2008). B. Comparing methods for handling missing values in food- frequency questionnaires and proposing k nearest neighbours imputation: effects on dietary intake in the Norwegian women and Cancer study (NOWAC). Public Health Nutr.

[42] The Danish Ministry of Education (2017). Rapport fra udvalget om ligestilling i dagtilbud og uddannelse.

[43] De Bourdeaudhuij I, Van Cauwenberghe E, Spittaels H, Oppert J, Rostami C, Brug J, Van Lenthe F, Lobstein T, Maes L (2011). School-based interventions promoting both physical activity and healthy eating in Europe: a systematic review within the HOPE project. Obes Rev.

[44] O'Loughlin J, Renaud L, Richard L, Gomez LS, Paradis G (1998). Correlates of the sustainability of community-based heart health promotion interventions. Prev Med.

