# Supplementary material for: The effect of introducing a free breakfast club on eating habits among students at vocational schools
Source: BMC Public Health. 2019 Apr 3;19:369. doi: 10.1186/s12889-019-6701-9 (PMC6446271; doi:10.1186/s12889-019-6701-9)
Supplement: Supplementary file 1 — Questionnaire to students The questionnaire concerns eating habits and students weekdays in school. (DOCX 88 kb) [file 12889_2019_6701_MOESM1_ESM.docx]

# Additional file 1 Questionnaire to students

**About you**

1. Your name

|  |
| --- |

2. You are

| Male |  |
| --- | --- |
| Female |  |

3. How old are you?

| Yonger than 15 |  |
| --- | --- |
| 15 – 19 |  |
| 20 – 24 |  |
| 25 – 29 |  |
| Older than 29 |  |

4. What line of education are you currently enrolled in?

| Car, aircraft and other transportation |  |
| --- | --- |
| Construction |  |
| Housing- and user service |  |
| Animals, plants and nature |  |
| Body and style |  |
| Food production |  |
| Media |  |
| Trade |  |
| Production and development |  |
| Electricity, automation and IT |  |
| Health care and pedagogy |  |
| Transport and logistics |  |
| Other |  |

5. What school do you go to?

|  |
| --- |

6. Which class do you go to?

|  |
| --- |

7. Have you been enrolled in other educations after primary school? (Other education may be another vocational school, high schools, The higher Commercial Examination Programme (HHX) The Higher Preparatory Examination (HF), The Higher Technical Examination (HTX), Social and Health Care Training Programme (SOSU) or agricultural schools)

| Yes |  |
| --- | --- |
| No |  |
| If yes which one? |  |

8. How do you live?

| Alone |  |
| --- | --- |
| With one or both of my biological parents |  |
| Other adults, specify |  |
| With girlfriend/boyfriend, spouse, and/or children |  |
| With a friend |  |
| Dormitory or community |  |

**Your transport to school**

9. How far is it to your school? (Km)

|  |
| --- |

10. How long does it take you to get to school? (Hours/minutes)

|  |
| --- |

11. What time do you usually leave home in the morning to go to school?

|  |
| --- |

12. How do you usually get to school?

| Drive a car |  |
| --- | --- |
| In car with friends |  |
| Walk |  |
| Bike |  |
| Bus and/or train |  |
| Moped/motorcycle |  |
| Other, specify |  |

**Your breakfast habits**

(N.B.: Breakfast is food or drink consumed before 9:00)

13. Think back of previous school-week, how often did you eat breakfast in that school-week?

| All 5 days |  |
| --- | --- |
| 3-4 days |  |
| 1-2 days |  |
| None of the days |  |

14. On the days you didn’t eat breakfast, on what hours did you consume your first meal?

| 9-10 AM |  |
| --- | --- |
| 10-11AM |  |
| 11-12 AM |  |
| I don’t eat breakfast |  |

15. The days you had breakfast during last school-week, where did you consume it?

| At home before school |  |
| --- | --- |
| I ate the lunch box on the way to school |  |
| I bought and ate breakfast on my way to school |  |
| I bought and ate in the canteen at school |  |
| I ate the breakfast served at school during the first class |  |

16. Think back of previous school-week, how often did you eat the following before 9?

|  | All 5 days | 3-4 days | 1-2 days | Didn’t eat |
| --- | --- | --- | --- | --- |
| Light bread or bun |  |  |  |  |
| Whole meal bread or rye bread |  |  |  |  |
| Oats |  |  |  |  |
| Cornflakes |  |  |  |  |
| Oat Cushions, Mini Oat Cushions etc. |  |  |  |  |
| Frosties, Coco Pops, Sugar puffs etc. |  |  |  |  |
| Muesli |  |  |  |  |
| Yoghurt, natural |  |  |  |  |
| Fruit yoghurt |  |  |  |  |
| Danish, cakes or crackers |  |  |  |  |
| Vegetables |  |  |  |  |
| Fruit |  |  |  |  |
| Other |  |  |  |  |

17. Think back of previous school week, what did you drink before 9?

|  | All 5 days | 3-4 days | 1-2 days | Didn’t drink |
| --- | --- | --- | --- | --- |
| Water |  |  |  |  |
| Milk |  |  |  |  |
| Coffee or tea |  |  |  |  |
| Cocoa |  |  |  |  |
| Juice |  |  |  |  |
| Smoothie |  |  |  |  |
| Drinking yoghurt |  |  |  |  |
| Diet soft drink, diet ice-tea, diet squash-drink |  |  |  |  |
| Soft drink, ice tea or squash-drink |  |  |  |  |
| Beer or other alcohol |  |  |  |  |
| Other |  |  |  |  |

18. Choose 3 main reasons for your choice of breakfast

| It’s fast and easy |  |
| --- | --- |
| It’s cheap |  |
| It tastes good |  |
| It looks good and appealing |  |
| It fills sufficiently |  |
| It’s something I’m in mood for |  |
| It’s healthy |  |
| That’s something I usually eat |  |
| It’s what I have available at home |  |
| Other, specify |  |
| I don’t eat or drink breakfast |  |

**Your mornings**

Mornings in this questionnaire are between 9 and 11

19. Think back of previous school-week, how often did you eat the following between 9 and 11?

|  | All 5 days | 3-4 days | 1-2 days | Didn’t eat |
| --- | --- | --- | --- | --- |
| Whole wheat or rye bread |  |  |  |  |
| Light bread or bun |  |  |  |  |
| Sandwich |  |  |  |  |
| Candy, chocolate or chips |  |  |  |  |
| Fast food (i.e. pizza, burger, hotdog, fries, shawarma) |  |  |  |  |
| Cakes, cookie or Danish |  |  |  |  |
| Fruit |  |  |  |  |
| Vegetables |  |  |  |  |
| Fruit yoghurt |  |  |  |  |
| Yoghurt, natural |  |  |  |  |
| Other |  |  |  |  |

20. Think back of previous school-week, how often did you drink the following between 9 and 11?

|  | All 5 days | 3-4 days | 1-2 days | Didn’t drink |
| --- | --- | --- | --- | --- |
| Water |  |  |  |  |
| Milk |  |  |  |  |
| Coffee or tea |  |  |  |  |
| Cocoa |  |  |  |  |
| Juice |  |  |  |  |
| Smoothie |  |  |  |  |
| Drinking yoghurt |  |  |  |  |
| Diet soft drink, diet ice-tea, diet squash-drink |  |  |  |  |
| Soft drink, ice tea or squash-drink |  |  |  |  |
| Beer or other alcohol |  |  |  |  |
| Other |  |  |  |  |

**Breakfast served at school**

21. How much do you agree on the following statements regarding breakfast at school?

|  | Completely agree | Mostly agree | Neither | Mostly disagree | Completely disagree |
| --- | --- | --- | --- | --- | --- |
| Breakfast during the first  Class of the day is a good idea |  |  |  |  |  |
| It’s good for fellowship with  Teachers and co-students to  Eat breakfast together |  |  |  |  |  |
| I feel more comfortable at school  When there is common breakfast |  |  |  |  |  |
| I talk more with my teachers when there is common breakfast at school |  |  |  |  |  |
| It is preferable that breakfast is served BEFORE school begins than in the first class |  |  |  |  |  |
| I don’t care about the social factor in eating breakfast with my classmates |  |  |  |  |  |
| It is a waste of time from school to serve breakfast to the students during class |  |  |  |  |  |
| I would rather use my time on curriculum than eating breakfast |  |  |  |  |  |
| I believe it is the students own responsibility to eat breakfast |  |  |  |  |  |

22. How much do you agree on the following statements regarding the breakfast program in school

|  | Completely agree | Mostly agree | Neither | Mostly disagree | Completely disagree |
| --- | --- | --- | --- | --- | --- |
| I’m wiling to pay 15 dk.kr a day for daily served breakfast at school |  |  |  |  |  |
| I’m wiling to pay 10 dk.kr a day for daily served breakfast at school |  |  |  |  |  |
| If I should pay for breakfast I want to decide which type of breakfast I eat |  |  |  |  |  |
| I think that school should pay for breakfast |  |  |  |  |  |

23. In your opinion, how important is it to eat breakfast?

| Very high degree | High degree | Somewhat | Less degree | Not at all |
| --- | --- | --- | --- | --- |
|  |  |  |  |  |

**Your weekday in school**

24. How much do you agree on the following statements regarding how you feel in school?

|  | Completely agree | Mostly agree | Neither | Mostly disagree | Completely disagree |
| --- | --- | --- | --- | --- | --- |
| I feel good in school |  |  |  |  |  |
| I get along well with my fellow students |  |  |  |  |  |
| I talk to my fellow students |  |  |  |  |  |
| I get along well with my teachers |  |  |  |  |  |
| I often to with my teachers |  |  |  |  |  |
| I can easily concentrate in class |  |  |  |  |  |
| There is too much noise during class |  |  |  |  |  |
| I participate in social activities at school |  |  |  |  |  |
| I’m tired of school |  |  |  |  |  |
| I consider dropping out of school |  |  |  |  |  |
| I feel lonely in school |  |  |  |  |  |

25. I often feel tired and lethargic in school

| Yes |  |
| --- | --- |
| No |  |

26. How much do you agree or disagree on the following statements regarding when you feel tired and lethargic in school?

|  | Completely agree | Mostly agree | Neither | Mostly disagree | Completely disagree |
| --- | --- | --- | --- | --- | --- |
| I most often feel tired and lethargic in the morning |  |  |  |  |  |
| I most often feel tired and lethargic in just before lunch |  |  |  |  |  |
| I most often feel tired and lethargic in just after lunch |  |  |  |  |  |
| I most often feel tired and lethargic in the afternoon |  |  |  |  |  |

**The last questions concern your overall eating habits**

27. How often do you eat the following?

|  | More than once a week | 5 – 7 times a week | 3 – 4 times a week | 1 – 2 times a week | 1 – 3 times a month |
| --- | --- | --- | --- | --- | --- |
| Cakes, ice cream |  |  |  |  |  |
| Fastfood (burger, shawarma, pizza, sausages etc.) |  |  |  |  |  |
| Snacks (chips, popcorn etc.) |  |  |  |  |  |
| Candy, chocolate |  |  |  |  |  |

28. How many pieces fruit do you eat?

| More than 6 a day |  |
| --- | --- |
| 5 – 6 a day |  |
| 3 – 4 a day |  |
| 1 – 2 a day |  |
| 5 – 6 a week |  |
| 3 – 4 a week |  |
| 1 – 2 a week |  |
| More rare or never |  |

29. How often do you eat the following?

|  | More than once a day | 5 – 7 times a week | 3 – 4 times a week | 1 – 2 times a week | 1 – 3 times a month |
| --- | --- | --- | --- | --- | --- |
| Rye bread |  |  |  |  |  |
| Whole meal crispbread |  |  |  |  |  |
| Whole meal bread |  |  |  |  |  |
| Oatmeal |  |  |  |  |  |
| Whole grain pasta or rice |  |  |  |  |  |
| Polenta, bulgur, wheat kernels |  |  |  |  |  |

**Thank you for your help**
